# Prevalence and associated factors of depression among patients with HIV/AIDS in Hawassa, Ethiopia, cross-sectional study

**DOI:** 10.1186/s12991-018-0215-1

**Published:** 2018-10-30

**Authors:** Bereket Duko, Epherem Geja, Mahlet Zewude, Semere Mekonen

**Affiliations:** 1 Faculty of Health Sciences, College of Medicine and Health Sciences, Hawassa University, P. O. Box 1560, Hawassa, Ethiopia; 2 Hawassa University Comprehensive Specialized Hospital, Hawassa University, Hawassa, Ethiopia

**Keywords:** Depressive symptom, Perceived stigma, Social support, HIV, Ethiopia, Prevalence, Associated factors, Depression, Hawassa, South Ethiopia

## Abstract

**Background:**

Globally, 350 million people are affected by depression and 800,000 people die due to suicide every year due to depression. People living with HIV/AIDS face different challenges, including HIV-related perceived stigma, lack of social support and also depression. This study aimed to assess prevalence and factors associated with depressive symptom among people living with HIV/AIDS attending Hawassa University Comprehensive Specialized Hospital, Hawassa, Ethiopia.

**Methods:**

Hospital-based cross-sectional study was implemented in 2016. A total of 401 HIV-positive patients who had regular visit at Hawassa University Comprehensive Specialized Hospital, Hawassa, Ethiopia were included in the study. Systematic random sampling technique was used to recruit study participants. Patient Health Questionnaire item nine (PHQ-9) was used to assess depressive symptoms. In addition to this, Oslo social support scale and HIV perceived stigma scale were used to assess social support and HIV-related perceived stigma, respectively.

**Results:**

A total of 401 study participants were included in the study, giving a response rate of 96.2%. The mean age of the respondents was 38 years (SD ± 10.23). This study revealed that 48.6% of HIV-positive patients had depression. Patients who had poor social support [AOR = 2.53, (95% CI 1.70, 9.13)], HIV-related perceived stigma [AOR = 2.83, (95% CI 1.78, 4.48)] and CD4 cell count < 200 [AOR = 3.89, (95% CI 1.02, 14.83)] were more likely to have depression as compared to individuals who had good social support, no perceived HIV stigma and CD4 cell count > 200, respectively.

**Conclusion:**

Having poor social support, HIV-related perceived stigma and low CD4 cell count (< 200) had statistically significant association with depressive symptom. Training of health workers in ART clinics and availing manuals on assessing mental health issues is useful to screen and treat depression among HIV patients.

## Background

HIV/AIDS is one of a chronic disease which affects human immune systems and it increases vulnerability to infections and other immunological disorders [1]. Globally, different studies in 2013 revealed that an estimated 35 million people were living with HIV/AIDS, of which 24.7 million are living in Sub-Saharan Africa and 1.6 million people died related to HIV/AIDS [2]. In developing countries, 9.5 million people were receiving HIV treatment in 2012 [3].

According to the WHO 2015 report, 350 million people were affected by depression worldwide. Due to this problem, over 800,000 people die by suicide every year globally [4]. WHO estimated that the incidence of suicide related to depression will reach approximately 1.53 million people by the year 2020. Based on finding from general population study, the life-time risk of depression is one in five women and one in ten men in their lifetime [5].

Findings from different studies show that 121 million people living with HIV/ADIS are affected by depression globally [6]. Studies conducted in different countries on prevalence of depression among HIV patients showed 58.75% in Delhi (India) [7], 29.4% in Brazil [8], 54.4% in Italy [9], 37% in United States [10], 25.4% in South Africa [6, 11], 25.3% of women and 31.4% of men in Botswana [12], 47% in Uganda [13], 43.9% in Mekele, Ethiopia [14], 45.8% in Harar, Ethiopia [15] and 38.94% in Debrebirhan, Ethiopia [16].

Depressive symptom among HIV-positive clients is associated with low income, widowed, being female, non-adherence of ART, having frequent of schedule for clinical visit in a month, low educational status, being female, age category (40–49), and having stage III and Stage IV HIV-related symptom [16, 17].

Being mentally impaired has been linked with an impaired adherence to ART and poor treatment outcome, decrease in CD4 count and increase in viral load. In addition, depression has been associated with high-risk behaviors like engaging in unsafe sex [11, 15, 17].

Based on different study findings, the magnitude of depressive symptom among people living with HIV/AIDS is high. Though it has a great impact on their treatment outcome, it was not assessed at Hawassa University Comprehensive Specialized Hospital. Therefore, this study aims to assess the prevalence and factors of depressive symptom among people living with HIV attending Hawassa University Comprehensive Specialized Hospital, ART clinic, South Ethiopia.

## Methods

### Study setting and population

Hospital-based cross-sectional study design was implemented from April to May 2016 at Hawassa University Comprehensive Specialized Hospital, Hawassa, Ethiopia. Among 1440 HIV patients who had regular follow-up at ART clinics, 417 study participants were recruited for the study; those unable to communicate because of their illness and those who need intensive care were excluded from the study. Study participants were included using systematic random sampling technique, *K* = 3. Sixteen patients were refused to participate in the study.

### Data collection

Trained and experienced nurses had collected the data using pretested interviewer administered questionnaire. The data collection tool includes socio-demographic characteristics (age, education, occupation, marital status and others). Oslo 3-item social support scale has the sum score scale ranging from 3 to 14 with three broad categories: “poor support” 3–8, “moderate support” 9–11 and “strong support” 12–14 [18]. It was reliable in our study (Cronbach’s *α* = 0.88). HIV-related perceived stigma was collected by an 11-item HIV stigma scale. It consisted of four-point Likert scale (strongly disagree, disagree, agree, strongly agree) questions concerning perceived isolation, shame, guilt and disclosure of the HIV status. The item scores of the stigma questions were summed to construct a single stigma variable. Our study participants were classified as having or not having perceived stigma using the mean of the stigma variable as cutoff point [19, 20]. The instrument was adopted and translated to Amharic language and back to English and highly reliable in the study (Cronbach’s *α* = 0.92). The presence of depression was assessed by patient health questionnaires item nine (PHQ-9). It is a 9-item questionnaire, commonly used to screen for symptoms of depression in primary health care and in outpatients and validated in Ethiopia with sensitivity = 86% and specificity = 67%. The scales use a cutoff score for depression of greater than or equal to 5 [21].

### Data processing and analyses

SPSS version 20 was used to analyze the data. The association of each independent variable with the outcome variable was seen by bivariate analysis. In order to identify potential confounders, binary logistic regression model was used. A *p* value of less than 0.05 was considered statistically significant and adjusted odds ratio with 95% CI was calculated to determine association.

## Results

### Socio-demographic characteristics of the study participants

A total of 401 study participants were included in the study, giving a response rate of 96.2%. The mean (± SD) age of the respondents was 38 years (± 10.228). Among the study participants, 149 (38.9%) were in age range between 35 and 44 years, 193 (50.4%) were orthodox religion followers, 178 (46.5%) were married, 138 (36%) were attended primary education, 96 (25.1%) were house wife, and 340 (88.8%) were living in urban. The median monthly income of the respondents was 875 Ethiopian birr (31.45 USD) (Table 1).

**Table 1:** Distribution of people living with HIV/AIDS at Hawassa University Comprehensive Specialized Hospital, Hawassa, Ethiopia, 2016/2017

Characteristics	Category	Frequency	Percent (%)
Sex	Male	129	29
	Female	272	71
Age	18–34	141	36.8
	35–44	149	38.9
	45–54	62	16.2
	> 54	31	8.1
Residence	Urban	340	88.8
	Rural	43	11.2
Religion	Protestant	160	41.8
	Orthodox	193	50.4
	Muslim	29	7.6
Educational level	Unable to write and read	68	17.8
	Primary education (grade 1–8)	138	36
	Secondary education (grade 9–12)	110	28.7
	Tertiary education (college and above)	67	17.5
Ethnicity	Sidama	48	12.5
	Oromo	88	23.0
	Amhara	93	24.3
	Wolaita	102	26.6
	Gurage	32	8.4
	Other	20	5.2
Marital status	Single	69	18.0
	Married	178	46.5
	Separated	19	5.0
	Divorced	45	11.7
	Widowed/widower	72	18.8
Occupation status	Merchant	76	19.8
	Government employee	65	17.0
	Privet employee	71	18.5
	Day laborer	33	8.6
	Student	17	4.4
	House wife	96	25.1
	Jobless	25	6.5
Monthly income	< 735ETB per month	199	52.0
	735–1176ETBper month	49	12.8
	> 1176ETB per month	135	35.2

### Clinical and psychosocial characteristics of the study participants

Among respondents, the maximum CD4 cell count was 1622 with a mean of 541.08. 330 (86.2%) of the study participants had CD4 cell counts ranges between 200 and 1000. 357, (93.2%) of respondents were on ART, 162 (42.3%) were found in stage II HIV/AIDS, 259(67.6%) had poor social support, 168 (43.9%) had perceived stigma and 72 (18.8%) were current substance (khat, alcohol, cigarette) users (Table 2).

**Table 2:** Description of clinical and psychosocial factors among people living with HIV/AIDS at Hawassa University Comprehensive Specialized Hospital, Hawassa, Ethiopia, 2016/2017

Variables	Category	Frequency	Percent %
CD4 cell count	< 200	33	8.6
	200–1000	330	86.2
	≥ 1000	20	5.2
Started ART taking	Yes	357	93.2
	No	26	6.8
Perceived stigma	Yes	168	43.9
	No	215	56.1
Current substance	Yes	72	18.8
	No	311	81.2
HIV/AIDS stages	Stage I	150	39.2
	Stage II	162	42.3
	Stage III	58	15.1
	Stage IV	13	3.4
Social support	Poor social support	259	67.6
	Moderate social support	110	28.7
	Strong social support	14	3.7

### Prevalence of depressive symptom among the study participants

Depressive symptom was found using PHQ-9 scale. Based on the cutoff point ≥ 11, 48.6% of the HIV clients had depression.

### Factors associated with depressive Symptoms

Binary logistic regression analysis revealed that poor social support, CD4 count (< 200) and perceived HIV stigma were associated with depressive symptom (Table 3).

**Table 3:** Factors associated with depression among people living with HIV/AIDS at Hawassa University Comprehensive Specialized Hospital, Hawassa, Ethiopia, 2016/2017

Characteristics	Depression		COR (95% CI)	AOR (95% CI)
	Yes	No		
Sex				
Female	140	132	1.49 (0.96, 2.34)	1.44 (0.82, 2.52)
Male	46	65	1	1
Age				
18–34	74	67	1.34 (0.61, 2.92)	1.31 (0.51,3.38)
35–44	62	87	0.86 (0.397, 1.885)	0.86 (0.34,2.14)
45–54	36	26	1.68 (0.71, 4.01)	1.59 (0.61, 4.18)
> 54	14	17	*1*	*1*
Educational level				
Unable to read and write	34	34	1.23 (0.63, 2.43)	
Primary education	74	64	1.43 (0.79, 2.26)	
Secondary education	48	62	0.96 (0.52, 1.76)	
Tertiary education	30	37	1	1
Marital status				
Married	88	90	1.52 (0.86, 2.67)	1.76 (0.89, 3.46)
Separated	7	12	0.91 (0.32, 2.59)	0.83 (0.24,2.87)
Divorced	22	23	1.48 (0.69, 3.17)	1.28 (0.52, 3.15)
Widowed/widower	42	30	2.17 (1.11, 4.27)	1.78 (0.72,4.38)
Single	27	42	1	1
Monthly income				
< 735 ETB	105	94	1.67 (1.07, 2.60)	1.60 (0.95, 2.68)
735–1176	27	22	1.84 (0.95, 3.56)	1.40 (0.67,2.95)
> 1176	54	81	*1*	*1*
Substance use				
Yes	37	35	1.13 (0.68,1.92)	
No	149	162	*1*	*1*
ART taking				
Yes	173	184	0.940 (0.42, 2.08)	
No	13	13	*1*	*1*
HIV/AIDS stages				
Stage II	74	88	0.94 (0.59, 1.46)	0.77 (0.46, 1.29)
Stage III	31	27	1.27 (0.69, 2.35)	1.02 (0.49, 2.10)
Stage IV	10	3	3.71 (0.98, 14.02)	2.79 (0.64, 12.09)
Stage I	71	79	*1*	*1*
Perceived stigma				
Yes	108	60	3.16 (2.08, 4.82)	2.83 (1.78, 4.48)**
No	78	137	1	*1*
Social support				
Poor	104	155	1.21 (0.39, 3.71)	2.53 (1.70, 9.13)**
Moderate	77	33	4.20 (1.31,13.48)	7.09 (1.91, 26.29)*
Strong	5	9	1	*1*
CD4				
< 200	23	10	2.30 (0.73, 7.25)	3.89 (1.02, 14.83)*
200–1000	153	177	0.86 (0.35, 2.13)	1.27 (0.47, 3.47)
≥ 1000	10	10	1	*1*

Depression (Yes)-PHQ ≥ 11, * significant association (*p*-value < 0.05) ** significant association (*p*-value < 0.01)

Italic values represent references of the variable

## Discussion

Institution-based cross-sectional study was conducted to assess the prevalence and factors associated with depression among patients HIV/AIDS at Hawassa University Comprehensive specialized hospital using PHQ9. The finding of this study (48.6%) was higher than studies in rural South Africa 42.4% [6, 11], in Malawi 18.9% [22], and in Ethiopia 43.9%, 45.8%, 38.94% in Mekele, Harar and Debreberihan, respectively [14–16]. On the other side, the study finding was lower than studies done in Delhi (India) 58.75% [7], North Central Nigeria 56.7% [23], in Cameroon 63% [24] and in Ethiopia [25]. The difference might be related to study design, data collection tool, sample size and study participant’s variation.

HIV-related perceived stigma had significant association with depressive symptom. The finding is similar to the study done in Botswana [12], in Ethiopia [14–16, 25]. Having HIV, which is one of the chronic life-long diseases and which is prone to high levels of stigma, they may find it easier to be alone to avoid stigma or discrimination, or they may not have the energy to be socially engaged [26].

Clients who had poor social support were 2.5 times more likely to have depressive symptom when compared to clients who had strong social support (AOR = 2.53, 95% CI 1.70, 9.13). The finding was similar to the study conducted in Delhi (India) [7], in Nigeria in 2008 [27], and in North Central Nigeria in 2013 [23]. This might be due to the fact that social isolation reduces social support, which can have a negative impact on mental and physical well-being [28].

Individuals who had < 200 CD4 cell count had significant association with depressive symptom. This was similar to the study conducted in Malawi [22], and Debrebirhan, Ethiopia [16]. This might be due to severe immune depression and HIV illness is underlining causes of depression [29].

Unlike other study, being female sex, being divorced and unmarried and those using substance had no statistically significant association with depression.

## Conclusion

Depressive symptom was high (48.6%) among the current study population. Perceived HIV-related stigma, poor social support and CD4 count (< 200) had significant association with depressive symptom. Hence, depression is highly prevalent among HIV-positive patients, still underdiagnosed and undertreated but it needs further research. Therefore, Ministry of Health should give more emphasis to those clients with depressive symptoms. Further research on risk factors of depression should be conducted to strengthen and broaden the current findings.

## Limitation of the study

We did not do detailed validation study for perceived HIV-related stigma scale and Oslo 3-item social support scale.
